# Navigating medical assistance in dying from Bill C-14 to Bill C-7: a qualitative study

**DOI:** 10.1186/s12913-021-07222-5

**Published:** 2021-11-04

**Authors:** Barbara Pesut, Sally Thorne, David Kenneth Wright, Catharine Schiller, Madison Huggins, Gloria Puurveen, Kenneth Chambaere

**Affiliations:** 1 School of Nursing, Principal Research Chair in Palliative and End-of-Life Care, University of British Columbia Okanagan, ARTS 3rd Floor, 1147 Research Road, BC V1V 1V7 Kelowna, Canada; 2 School of Nursing, University of British Columbia, BC V6T 2B5 Vancouver, Canada; 3 School of Nursing, University of Ottawa, Ontario K1H 8M5 Ottawa, Canada; 4 School of Nursing, University of Northern British Columbia, 3333 University Way, BC V2N 4Z9 Prince George, Canada; 5 School of Nursing, University of British Columbia Okanagan, ARTS 3rd Floor, 1147 Research Road, BC V1V 1V7 Kelowna, Canada; 6 Public Health, Sociology & Ethics of the End of Life, End-of-Life Care Research Group, Ghent University & Vrije Universiteit Brussel (VUB), C. Heymanslaan 10, B-9000 Ghent, Belgium

**Keywords:** Medical assistance in dying, End of life, Nursing, Qualitative, Legislation, Healthcare systems

## Abstract

**Background:**

Even as healthcare providers and systems were settling into the processes required for Medical Assistance in Dying (MAID) under Bill C-14, new legislation was introduced (Bill C-7) that extended assisted death to persons whose natural death is not reasonably foreseeable. The purpose of this paper is to describe the experiences of nurses and nurse practitioners with the implementation and ongoing development of this transition.

**Methods:**

This qualitative longitudinal descriptive study gathered data through semi-structured telephone interviews with nurses from across Canada; cross sectional data from 2020 to 2021 is reported here. The study received ethical approval and all participants provided written consent.

**Findings:**

Participants included nurses (*n *= 34) and nurse practitioners (*n *= 16) with significant experience with MAID. Participants described how MAID had transitioned from a new, secretive, and anxiety-producing procedure to one that was increasingly visible and normalized, although this normalization did not necessarily mitigate the emotional impact. MAID was becoming more accessible, and participants were learning to trust the process. However, the work was becoming increasingly complex, labour intensive, and often poorly remunerated. Although many participants described a degree of integration between MAID and palliative care services, there remained ongoing tensions around equitable access to both. Participants described an evolving gestalt of determining persons’ eligibility for MAID that required a high degree of clinical judgement. Deeming someone ineligible was intensely stressful for all involved and so participants had learned to be resourceful in avoiding this possibility. The required 10-day waiting period was difficult emotionally, particularly if persons worried about losing capacity to give final consent. The implementation of C-7 was perceived to be particularly challenging due to the nature of the population that would seek MAID and the resultant complexity of trying to address the origins of their suffering within a resource-strapped system.

**Conclusions:**

Significant social and system calibration must occur to accommodate assisted death as an end-of-life option. The transition to offering MAID for those whose natural death is not reasonably foreseeable will require intensive navigation of a sometimes siloed and inaccessible system. High quality MAID care should be both relational and dialogical and those who provide such care require expert communication skills and knowledge of the healthcare system.

**Supplementary Information:**

The online version contains supplementary material available at 10.1186/s12913-021-07222-5.

## Background

Medical assistance in dying, referred to in Canada as MAID, has been evolving at a rapid rate. First introduced to the House of Commons as the subject of Bill C-14 (*An Act to Amend the Criminal Code and to Make Related Amendments to Other Acts [Medical Assistance in Dying], 2016*), it received Royal Assent on June 17, 2016 [1]. Even as Bill C-14 was being implemented, there was ongoing and robust debate about whether the law had gone far enough (see Table 1 for eligibility and safeguards).

**Table 1:** Select eligibility requirements and safeguards

Bill	Eligibility	Safeguards
Bill C-14	(1) A serious and incurable, illness, disease or disability; (2) In an advanced state of irreversible decline; (3) Intolerable physical or psychological suffering that cannot be relieved under conditions that the patient deems acceptable; and (4) Where natural death had become reasonably foreseeable, without a prognosis having been made about length of time remaining.	(1) Requests had to be made in writing and signed by two independent witnesses; (2) 10-day waiting period that could only be waived if the applicant was at risk of losing capacity; (3) Persons had to provide final consent immediately before the injection that would cause death
Bill C-7 Natural death reasonably foreseeable track.	Final consent could be waived as long as (1) they met all of the eligibility criteria; (2) they entered into an agreement in writing that included a specific day for the MAID procedure; (3) they were informed of the risk of losing capacity; (4) in the written agreement they consented to having MAID on that day if they had lost capacity; and (5) at the time of administration they do not demonstrate refusal or resistance to have the substance administered.	Request signed by one independent witness. No 10-day waiting period
Bill C-7 Natural death not reasonably foreseeable track.	Final consent can be waived if the person has self-administered and lost the capacity to consent but has not died, if the following conditions are met. Before the person lost the capacity to consent to MAID, the person entered into an agreement in writing with the physician or NP providing MAID that • requires the physician or NP to be present at the time of the self-administration; and • allows the physician or NP to administer a second substance to cause the person’s death if the person lost capacity to consent and did not die within a specified period after self-administration. The person self-administered the first substance but did not die within the specified period and has lost capacity to consent to MAID. The second substance is administered to the person in accordance with the terms of the arrangement.	Request signed by one independent witness There are at least 90 clear days between the day on which the first assessment begins and the day on which medical assistance in dying is provided, or – if the assessments have been completed and both of the physicians or NPs are of the opinion that the loss of the person’s capacity to provide consent to receive medical assistance in dying is imminent – any shorter period that the physician or NP who is to provide MAID considers appropriate in the circumstances.

Much of the early debate about MAID in Canada focused on what it meant for natural death to be reasonably foreseeable and whether such a requirement was even constitutional [2–4]. Questions remained about whether MAID should be allowed for mature minors, whether advance requests for MAID should be allowed, and whether eligibility should be extended to those whose sole underlying medical condition was a mental disorder. Expert reports were commissioned on each of these topics at the same time that MAID was legalized [5–7].

MAID rapidly became an end-of-life option for many Canadians. By the end of 2020, 21,589 Canadians had chosen to receive MAID, with the number of cases growing by 34.2 % between 2019 and 2020 [8]. In 2020, MAID accounted for 2.5 % of all deaths in Canada; however, this percentage was variable across geographic contexts. The percentage of deaths attributed to MAID varied from 0.9 to 4.0 % in 2020 [8] with one geographic area citing a rate as high as 4 % of all deaths by the end of 2018 [9].

Even as healthcare providers and systems were settling into the processes and requirements of Bill C-14, four separate court challenges were initiated on the grounds that Bill C-14 violated the *Canadian Charter of Rights and Freedoms* [10]. Finally, in 2019, the Superior Court of Quebec ruled that it violated the *Canadian Charter of Rights and Freedoms*[11] to restrict eligibility for MAID only to those persons whose natural deaths had been deemed reasonably foreseeable [10]. The plaintiffs in this case, Truchon and Gladu, were living with disabilities that made them eligible for MAID in every aspect except that their natural deaths were not reasonably foreseeable. The Federal government chose not to appeal this decision and instead began widespread consultation in the form of an online survey and expert roundtables in preparation for revisions to Bill C-14. The online survey resulted in an unprecedented 300,140 responses from the public, including 254,000 narrative comments. A content analysis of these comments indicated that, although there was widespread support of MAID, a portion of the Canadian population remained concerned about MAID and its evolution. Of the eight most common themes generated by the qualitative responses, two were about opposition to MAID or specific concerns about MAID [12]. Nevertheless, this broad-based consultation provided important information for the legislative revision to be known as Bill C-7.

Bill C-7 received Royal Assent on March 17, 2021, five years after the implementation of MAID through Bill C-14. The key change in Bill C-7 that is relevant to this paper is that a two-track system was created, one for those individuals whose natural death was reasonably foreseeable and a separate track for those whose death was not. Table 1 outlines those eligibility criteria and safeguards that were put into place for each track in this system that are relevant to this paper. Another important change was the ability to waive the final consent requirement for those persons whose natural death was reasonably foreseeable [10]. This change arose because of concerns that persons were choosing to undergo MAID earlier than they would have otherwise because of fears of losing the capacity to consent.

Several concerns were noted in the legislative summary of Bill C-7 [10]. First, there were concerns that MAID assessors would have difficulty deciding which track to assign persons to. Second, there was no timeframe outlining what constituted a reasonably foreseeable natural death. Third, there were moral concerns raised about providing MAID to those whose natural death is not reasonably foreseeable; some speculated that the resulting ethical, emotional, and psychological burden might result in fewer physicians and nurse practitioners being willing to assess for and provide MAID. Fourth, there were concerns that no time limit had been placed on the waiver of final consent. Theoretically, one could set a MAID date far into the future and then use this waiver as a form of advance consent.

This rapid development of MAID has generated significant debate in the palliative care community. In the context of MAID, key palliative care stakeholders have strongly advocated for universal access to high quality palliative care [13]. A frequently cited statistic is that less than 30 % of Canadians have access to high quality palliative care [14], although a recent report from the Canadian Institutes for Health Information suggested that it is difficult to obtain good information on palliative care accessibility, in part because of its variability across the country [15]. However, the second annual report on MAID in Canada indicated that 88.5 % of MAID recipients in 2020 had access to palliative care if needed [8]. Some have been concerned that the cost savings generated by MAID may influence the development of MAID programs over palliative care programs. A cost analysis of MAID in Canada suggested that annual healthcare spending could be reduced between 34.7 M and 138.8 M as a result of MAID, which far exceeds the anticipated costs of 1.5 to 14.8 M to run MAID programs [16]. To respond to these concerns, the Federal government committed to improving palliative care in Canada and developed a framework to guide that improvement [17]. A recent blog from the Canadian Parliamentary Budget Office indicated that, conservatively estimated, the Federal government invested 184 M between 2016 and 2020 [18], an amount that exceeds the cost savings generated by MAID, specifically toward palliative care services.

A review of empirical evidence from the Canadian context post-MAID implementation provides insight into significant factors influencing early experiences. From a systems perspective, many were struggling to set up processes that ensured patient-centered care and accessibility [19], particularly in light of the limited numbers of assessors and providers available [20], and the heavy workloads on those who were willing and able to provide MAID services [21]. Accessibility to MAID was influenced by the sometime contentious relationship between those care providers involved in MAID and those in palliative care [19, 22, 23]. There was a need to support healthcare providers involved in MAID in light of the emotional impact [20, 24, 25] and a need to provide healthcare providers with the knowledge and skills to assist with, or to assess and provide, MAID, particularly in light of vague eligibility criteria [20, 26, 27]. There was also a need to manage the relational challenges that arose between those who saw MAID as an acceptable moral option and those who did not [19–21, 25, 26]. Caring for families throughout the process and into bereavement has consistently been one of the most challenging aspects of MAID [20, 28, 29]. Given the potential impact of these factors on the quality of care provided through MAID, and on the ongoing debate about the relationship between MAID and palliative care, it is important to understand how MAID is evolving over time within a larger systems perspective.

Nurses are an important access point for understanding those factors that influence MAID implementation because (1) they lead the coordination services that are the primary point of access for patients; (2) nurse practitioners were given the legal authority under Bill C-14 to independently act as both assessors and providers of MAID in Canada; and (3) nurses are the healthcare providers who spend the most time with patients and families as they progress through the many phases of MAID contemplation, assessment, and provision. Thus, nurses have a vantage point on system issues that few others have access to. Therefore, the purpose of this paper is to describe the experiences of nurses and nurse practitioners with the implementation and ongoing development of MAID from Bill C-14 to Bill C-7.

## Methods

### Design

The design is a longitudinal qualitative study. Data collection is occurring at three time points: 2018/2019, 2020/2021, 2022/2023. This paper reports on a subset of cross-sectional data collected during between September 2020 and June 2021, a time when Bill C-7 was under review and then achieved early implementation. This study underwent ethical review by the behavioural research ethics board at the University of British Columbia Okanagan.

### Context

In Canada, MAID is often coordinated through centralized services that cover a geographic area. Clients can contact these services directly without a healthcare referral. The initial point of contact is a MAID coordinator who answers the initial inquiries, does an intake, and determines whether the client meet criteria for MAID assessment. The coordinator is responsible to find healthcare providers to conduct assessments and provision; these healthcare providers may not have a prior relationship with the patient. Some of these healthcare providers travel long distances to perform MAID provisions; although in the context of COVID-19, assessments can be conducted virtually.

### Sample

Previous participants who had initially been interviewed in 2018/19 were contacted by email. New participants were recruited using purposive and snowball sampling. Recruitment bulletins were distributed through national nursing agencies and through the Canadian Association of MAID Assessors and Providers. Strategies included email, Facebook, Twitter and word of mouth. We recruited from every English-speaking province; however, we did not recruit from the three territories in Canada as there were few reported cases of MAID during this time.

### Data collection

Data collection occurred through semi-structured telephone interviews. Telephone interviews were necessary to access a pan-Canadian sample. Interview questions probed around general experiences with MAID since the implementation of Bill C-14; perceptions of the developing relationships (or lack thereof) between MAID and palliative care systems; and the practical implementation of the eligibility criteria and safeguards, including those developing under Bill C-7. Interviews were audio-recorded, transcribed verbatim, checked for accuracy, and downloaded in NVIVO (QSR) for analysis.

### Data analysis

Coding was conducted inductively using the process consistent with Interpretive Description [30]. Two investigators began by co-constructing open codes which were then refined using analytic insights from three additional investigators. Constant comparative analysis was used to compare and contrast data to identify patterns and commonalities [31]; codes were further refined through this process. Thematic patterns were then developed and a narrative account was constructed. This narrative account was further developed and refined with input from all team members.

## Results

The sample consisted of 50 nurses and nurse practitioners, 26 of whom had been interviewed previously in 2108/2019 and 24 of whom were new participants interviewed in 2020/2021 (See Table 2).

**Table 2:** Participant characteristics (*N *= 50)

	N	%
**Ethnicity**		
Caucasian	45	90
Other	5	10
**Religious/Spiritual**		
Religious and Spiritual	24	48
Spiritual not Religious	17	34
Not Religious or Spiritual	9	18
Religious not Spiritual	0	0
**Primary population**^a^		
Specialized Palliative	26	52
Primary	17	34
Med Surg	13	26
Other	11	22
**Location of work**^a^		
Community	24	48
Hospital	18	36
Homecare	19	38
Hospice	12	24
Long-Term Care	10	20
Other	4	8
**Province worked in**		
British Columbia	23	46
Ontario	21	42
Other	6	12
**Sphere of work responsibilities**^a^		
Urban (>10,000)	41	82
Rural (<10,000)	28	56
Remote	8	16
**Nurse Designation**		
Registered Nurse	30	60
NP	16	32
Clinical Nurse Specialist	2	4
LPN	1	2
RPN	1	2
Mean Years in Professional Designation (mean ± SD)	17.9 (± 11.8)	
**Number of MAIDs Since Legalization**		
0-4	8	16
5-9	7	14
10-14	6	12
15-19	7	14
20-24	4	8
25 or more	18	36
Contentious objector to MAID		
Yes	5	10
No	45	90

^a^Responses are not mutually exclusive, multiple ‘yes’ responses possible

Themes developed from these interviews interpret the transitions and challenges associated with implementing Bill C-14; evolving tensions and synergies between MAID and palliative care; experiences of determining eligibility under Bill C-14; and challenges under the new Bill C-7.

### Implementing Bill C-14: transitions and challenges

Participants described how, in just a few short years, MAID had transitioned from a new, secretive, and anxiety-producing procedure to one that had become increasingly visible and normalized. With this normalization, they noted an increased openness about the procedure and an increased trust on the part of nurses that MAID assessors and providers were fulfilling their requirements under the law. These transitions paralleled an increased number of cases, an increase in the complexity of cases, and a subsequent effect on workload and funding.

#### Normalized to a point

Participants described what it was like to integrate MAID into their practice in the early days of Bill C-14. “*In the beginning, because everything was so new and big, like, MAID is really big and it’s still really big but it’s not as big.” (P6)* The perception of MAID as a major practice change was related to the novelty of the procedure relative to conventional end-of-life care, the political and ethical debate that had characterized legalization, and more importantly, the harsh reality that the procedure resulted in the rapid death of the patient. However, although participants spoke of the procedure itself becoming normalized, they often observed that the emotional impact never became normalized, and that this was a good sign. One nurse who had participated in numerous provisions since legalization said, “*I expect people to say that I am getting hardened to the whole thing, where it is just part of what you do in a day. But I say to them, ‘I’m never going to be hard to this and if I am, I can’t do it anymore*.’” (P40)

 Participants described work contexts that were increasingly characterized by acceptance and relaxation of the tensions associated with MAID, while acknowledging that the impact of it on patients and families still created an emotionally-laden environment. “*I’m more familiar, the doctors seem more familiar and more relaxed. It’s just calmer on the unit. The families and the patient are still… I mean, it’s still just a big thing if you ever go near the room or into the room or contemplate saying goodbye, those things are all still big.” (P6)* Normalizing the procedure of **MAID** therefore changed, but did not necessarily mitigate, the emotional impact.

#### From secrecy to visibility

Part of the normalization was a greater openness to talking about MAID. Participants described the secrecy that had characterized the early days of MAID implementation. Healthcare team members would not necessarily have known which of their patients were getting MAID until after it had occurred. Team members had been reluctant to talk to one another about MAID, because it was so morally divisive.

*Two years ago, I didn’t tell people that I did Medical Assistance in Dying because I did not want to defend my position. And I didn’t want somebody to defend theirs. Now I certainly don’t advertise it, but if it comes up in a conversation and I think that the person who is speaking would be open to knowing that I do that, then I will say I am part of the movement. (P14)*

The idea of being part of a “movement” was not an uncommon one as participants saw part of their mandate as making MAID acceptable to more patients and healthcare providers.

Workplace policies and practices had also changed, making MAID more visible. “*It’s on the calendar now that so-and-so’s being assessed for MAID and on our board in hospice, we’ve got all kinds of little tick marks for procedures, and MAID is one of them.” (P68)* Participants suggested that this openness was supported by first-hand accounts in the media about what it was like to live with suffering and the relief that the option of MAID provided. “*I find that a lot of the change in peoples' perspective has been not from arguments or the facts or the criteria or the law, it’s been the narratives. It’s been peoples' stories and peoples' experiences.” (P81)*

#### Greater accessibility

This increasing comfort and openness coincided with greater MAID accessibility. Initially, it could be difficult for MAID coordinators to find physicians or nurse practitioners who were willing to perform the assessments and provisions. “*In the initial stages of MAID there were certainly a number of physicians that did not want to be involved.” (P15)* However, at the time of these interviews, more providers were becoming involved. “*I have seen more providers in the community which is really good. There’s been times in the past where there were no providers, some hospitalists are coming from hospital to provide that service*.” *(P11)* As this participant indicated, the use of hospitalists for MAID assessments and provisions was one way to fill the gap of an insufficient number of providers willing to do provisions in community.

#### Trusting the process

This increase in accessibility was accompanied by an increased trust by nurses in assessors and providers. This trust was important, because nurses’ contributions to patient care throughout the decision and implementation processes were essential. As self-regulating professionals, they too were required to ensure that eligibility and safeguards had been addressed prior to their participation. “*As a registered nurse, it’s just always been my philosophy to do my due diligence. I never just go in and participate in a MAID because someone told me to.” (P73)* However, it was not always easy for nurses to get access to the documentation that allowed them to check eligibility and safeguard information. As a result, trust in those they were working with was essential. A nurse described the development of that trust.

*In the beginning I had the perspective of I’m going to go in there and I’m going to protect that patient and I’m going to make sure that they’re getting this MAID because they want it and because it’s legal and because it’s right. Whereas, by the end of it, I was, like, nope. I knew more, and I trusted the providers because I knew the providers. (P63)*

#### Increased cases, complexity, and workload

The increased volume in MAID cases, along with a concurrent need to develop systems and processes, meant that in some regions workload quickly became unmanageable. In one geographic region, the percentage of MAID deaths had increased from 2 to 4 % of all deaths within a one-year period. This meant working overtime to keep up with the demand. In other geographic areas, waitlists for assessment were developing and providers were having to group several provisions in a single day to keep up, a situation that was not considered best-practice, either for patients or providers. “*So, our patients were told, ‘Yes, you are eligible. If you want to have MAID, you will be scheduled in on Wednesday, July the whatever at 10 o’clock.’ The provider went from one person’s home to another and did four provisions in one day.” (P63)*

Nurses who were involved in MAID leadership positions (e.g., MAID coordinators) were required to perform multiple roles such as providing public and healthcare provider education; screening and coordinating of requests; developing policies and standards; and participating on committees at multiple decision-making levels. However, one of the most challenging and labour-intensive parts of their role was case management. Coordinators received initial inquiries from the public and were responsible to determine whether those cases should be referred to an assessor. Participants explained how after the media stories of the Court decision in which the eligibility criterion of reasonably foreseeable natural death was struck down, persons whose natural deaths were not reasonably foreseeable started contacting MAID coordination services about their eligibility. As these potential MAID candidates told the coordinators their stories of illness and suffering, it would often become apparent that they had not accessed important services that could alleviate that suffering. These coordinators were then ethically obligated to navigate them toward those services. “*I’m actually working with a case manager and it’s much beyond what would be the expectation of a MAID assessor normally; however, it is my obligation to make sure that the patient has been made aware of all of their care options.” (P30)*

MAID coordinators who were required to perform this navigation without the support of case managers encountered numerous barriers such as an inability to get sufficient patient information, a lack of services to which they could refer clients, a delay in the availability of those services, and reluctance on the part of some physicians to work with them because of their affiliation with MAID care. For example, a participant described trying to get information from a physician about a MAID applicant. “*There could be all kinds of levels of why he didn’t want to talk to me but clearly, he didn’t want to talk to me and he wasn’t going to share any information.” (P30)* All of this resulted in increased workloads for these coordinators.

#### Remuneration challenges

This type of intensive case management work consumed many hours, and participants suggested that the money available for MAID services was not keeping pace with demand. Remuneration could be problematic at both the system and individual level. At the time of this data collection, nurse practitioners in parts of the country were having difficulty being remunerated for their MAID work unless it was part of their regular job. As a result, some volunteered their time to do this work.

At a system level, participants cited the complex optics of giving more money to MAID. At the time that MAID was implemented in Canada, substantial new healthcare funding was being targeted toward improving palliative care in Canada, and this money could not be re-routed to support MAID.

*In the last year, palliative care was granted millions of dollars in our area, but, I need help, our little program (MAID) needs help, needs some more people. And we’re continually told ‘no, the money has been earmarked for Palliative Care and it cannot go for MAID, it cannot, cannot, cannot’ and I find that disturbing because the people that we are seeing with assisted dying are… not all of them but the high majority are the same people who would be eligible for Palliative Care. (P75)*

Thus, despite the increasing workload associated with MAID, providing more money for MAID programs could be interpreted as taking money away from palliative care even though public monies for healthcare in Canada are not constrained in this way. This interpretation would be problematic because the infusion of money into palliative care was meant to assure the public that persons would not choose MAID because of lack of palliative care services.

### MAID and palliative care: tensions and synergies

The funding optics described above were indicative of a number of concerns shared by study participants about the emerging relationships between MAID and palliative care. Many reflected on the impact on patient choice occasioned by inequities between the programs. Participants further spoke of the benefits and challenges of integrating MAID within palliative care systems of care.

#### Patient choice and inequities in access

The legalisation of MAID through Bill C-14 had an unanticipated positive impact upon palliative care, primarily in raising the profile of palliative care and calling attention to advance care planning as a priority direction. The public attention to MAID, and the tensions between MAID and palliative care that often played out in the public domain, served to raise peoples' awareness about end of life and palliative care overall.

*By virtue of the tension between the palliative care community and MAID, and there is that tension, what that did was shone a light on palliative care. It really raised the bar and raised awareness around what is palliative care, and raised a lot of questions. Why would somebody choose an assisted death versus palliative care options and services and what that might look like? (P80)*

Bringing these options to public attention proved fertile ground, and a sense of urgency around advance care planning ensued. And over time, as the participant’s accounts suggested, the tensions between MAID and palliative care were finally settling into a sense that informed patient choice was the unifying factor between the two.

*There was a politicization between palliative care and MAID and the cry for funding and I don’t hear that so much now anymore and I think it’s all quite valid to say that we want people to really make sure that they know what their palliative options are but then, the choice belongs to them. (P64)*

However, the availability and accessibility (or lack thereof) of MAID and palliative care services created ethical concerns about patient options and choice at end of life. Participants described how hard they tried to ensure that patients were aware of their various end-of-life options and that they were informed of these choices in an unbiased way. As one explained, “*I review all the available care options at end-of-life without bias and don’t talk about palliative care for 35 minutes and talk about MAID for five. Like, you have to give equal airtime to all of the options so that the patient doesn’t have an unconscious bias towards one.” (P4)* This participant was recognizing the importance of not emphasizing one option over another so as not to unduly influence client decision-making. Another participant suggested, “*If we’re coming in a principled and an ethical way, there should be no coercion, it should be a neutral giving of information for people to weigh up and make their own decisions and preferences*.” *(P64)*

But participants questioned whether it was possible to fulfill this obligation of choice when the options were bounded by what was available and accessible, given the funding limitations that characterized both MAID and palliative programs. Despite the recent infusion of funding for palliative care, there remained gaps across the country including healthcare provider knowledge of palliative care, services available in rural and remote communities, and a reluctance of primary care providers to introduce the idea of palliative care. Others described inequities between MAID and palliative care funding that influenced which services were more accessible to patients.

*MAID prescribers were being funded to travel to provide an assisted death, whereas our palliative care physicians, who have been doing home-based palliative care travelling to rural and remote areas, their time and travel were not compensated. There was just this real frustration. You are making MAID far more accessible than good palliative care*. *(P80)*

A prevailing idea that MAID must be accessible because it is legal and funded under the Canada Health Act was particularly challenging for some to accept if the same premises were not equally applied to palliative care. Accessibility to MAID, as evident in increasing numbers of cases, was sometimes seen as a positive program outcome. “*I remember this senior leader saying to me, ‘we’re really proud of our success rate with MAID.’ And I said, ‘Well, what do you mean by that?’ ‘Well, we’re really proud of our particular area and that whoever wants MAID, gets it, and we have one of the highest rates of MAID compliance.’” (P82)* In contrast, this participant suggested that disproportionately high MAID rates should trigger a deeper exploration of the reasons, including the possibility that the region was offering insufficient access to palliative care.

#### Benefits and challenges of program integration

The degree of integration between MAID and palliative care varied across Canadian contexts: from no integration, to an informal consulting relationship, to full clinical program integration, although it is important to note that even programs that reported high degrees of integration might still have separate organization reporting and financial structures. This meant that, even though the spirit of integration was followed, the mandatory separation enshrined in the legislation between MAID and palliative care remained.

Participants who had been involved in system-level integration between palliative care and MAID described the benefits and process of that integration. *“We really tried to push and shift the dialogue from it’s either palliative care or MAID to one that says it’s palliative care all the way through your experience. How you died doesn’t really matter.” (P80)* Despite the moral or philosophical objections that palliative clinicians might have toward MAID, there was acknowledgement that both were caring for the same population. Further, it was acknowledged that palliative care clinicians had unique and necessary expertise to contribute to the MAID process.

*For right now, our population is the same. And so, it makes sense that MAID is part of the palliative and end-of-life program, that we have more integration and collaboration with our MAID prescribers and our palliative care teams because our palliative care clinicians and teams are the best people to be having those difficult conversations with individuals around suffering. (P80)*

Another benefit of integration was the ability to build policy across programs that would benefit patients. For example, one participant described how the MAID and palliative teams working together developed policies that would ensure compassionate care across the spectrum. An example of this was a “kindness” policy in which any person desiring MAID who needed to be transferred out of a palliative facility for the procedure would not be asked to bear the cost of that transfer.^1^*(P75)*

This type of cooperation between the MAID and palliative programs was best achieved through relational processes. For example, this participant involved in integrating MAID with palliative care speaks of the slow and patient process of building relationship.

*I would say from day one, this relationship of being embedded in palliative care has been such a push, it’s a building of relationships. We need to be here, we want to be here, but just a gentle continual knock, knock, we’re here, awareness. Right? Very kind, very respectful of building relationships, building engagement, doing education, so we have worked super, super hard for that success. (P75)*

However, this relationship building required funds to bring self-employed physicians together for this education and relationship-building, funds that were not always available on an ongoing basis.

Despite the benefits of program integration, some participants remained highly cautious about the idea of fully integrating the MAID and palliative programs, and argued that it was important to keep the programs separate, while learning to share expertise.

*Well, I don’t know about full integration, to be honest. I think there’s still a discomfort there but I think there needs to be very strong links. I think it behooves palliative clinicians to understand MAID, its current directions and trends and issues, and understand how the MAID system works. But on the other hand, I think it’s really important that MAID providers have a strong working knowledge of palliative care and don’t come in with blinders thinking, you know, this is what I do and this is all I do and not needing to know more. I think we both serve the individual better if we have a full working knowledge of each other’s worlds. (P64)*

Participants spoke to the important expertise that palliative practitioners have around relieving suffering and having difficult conversations, skillsets they felt were essential to determining eligibility for MAID and ensuring that persons have the best quality of life for as long as reasonably possible.

*Our palliative care clinicians and teams are the best people to be having those difficult conversations with individuals around suffering. And they are the ones that can work with that whole person’s sense of suffering, not just the physical. I think the concern has always been, and certainly we have seen this, is that without palliative care, without individuals being touched by palliative care and having palliative care as part of their experience around, you know… what are their goals and wishes and how do they want to die? If they just go to ‘I’m suffering and I want it to end, give me an assisted death’, they lose out on a whole lot of opportunities by not having palliative care involved. (P80)*

The final drawback was that of workload. Nurse practitioners who were the most responsible practitioners for a group of palliative clients spoke of how the highly labour-intensive work of MAID could quickly overwhelm their practice, taking time away from their palliative clients. As a result, some had agreed only to be involved with MAID if it involved their own clients.

### Eligibility and safeguards under Bill C-14

The legal language of Bill C-14 required interpretation for clinical practice. Participants described how their understandings of the eligibility criteria had evolved, how stressful it was to determine that someone was ineligible, and how particularly resourceful persons were able to achieve their goal of having MAID even if they had been deemed ineligible. The most challenging safeguards to negotiate were the ten-day waiting period and the requirement of final consent.

#### Evolving gestalt of eligibility

When questioned about their experiences with MAID eligibility and safeguards, participants described an evolving understanding. “*I’ve noticed that the eligibility has loosened up or lightened up a little bit. I will say that in the past four years, I have probably seen more people qualify.” (P40)* This evolving understanding was necessary because, although the criteria and safeguards were clear in the legislation, the legal language was ambiguous and required clinical interpretation.

*‘Reasonably foreseeable’ is something that lawyers say all the time. Lawyers are very comfortable with that, they love the ambiguity of it, the flexibility of it, because it means you can argue about it, about what that means, and it creates a certain flexibility to the law but of course, as a provider or as a clinician, you don’t want flexibility, you don’t want ambiguity, you want certainty. (P81)*

Doing assessments on persons who did not clearly meet the eligibility criteria, or whose eligibility was “very grey” *(P67)*, required considerable clinical judgement, a process that one nurse described as a “*gestalt of the experience of the person*.” *(P76)* This gestalt was easier if the assessor knew the patient.

*When I’m assessing my own patient, I have a historical context, I know the dynamics, I know the diagnoses, I know that they’ve been offered appropriate palliative care, for example, I know where they are in their kind of cancer journey and so the assessments are much easier to do in terms of eligibility because I just have the historical background for the patient. (P94)*

Assessors learned from one another as they developed this understanding of the criteria. “*We have to be able to reflect on cases, have discourse, learn, just like with any other complex situation*.” *(P76)* This process entailed recognizing that there could be differences in clinical judgement about who should be eligible and that patients might demonstrate varying levels of determination to have MAID for different clinicians. These complex processes prompted much self-reflection. For example, this assessor was involved in a situation where there was conflicting information about what the patient wanted. “*It was quite obvious to me he wanted the MAID. And so, there I was pretty conflicted because I thought, ‘Am I trying to force this MAID because I know how it goes and it’s just a quick easy clinical thing? Am I missing something’”? (P63)*

The possibility that two providers might come to a different conclusion about whether a patient was eligible for MAID raised issues of equity.

*From an ethics perspective, you want consistency, you want similar cases to be treated similarly. So, if you get similar cases, so the same patient, same background, same circumstances with two different providers and one of the providers said, “Yes, you qualify”, and the other provider said, “No, you didn’t”, that is an issue of fairness, that is an issue of consistency, and that is not something we want in a program like MAID*. *(P81)*

Further, participants indicated there was still a lack of knowledge among healthcare providers and the public about the eligibility criteria and safeguards or about the process. Participants explained that physicians who were not acting as assessors and providers might be unaware that they did not have authority to deem someone ineligible. *“There’s this massive running gap between physicians about what their role is, how this actually works, what the eligibility criteria are, and we’re still seeing that four years later.” (P86)*

The striking down of the reasonably foreseeable natural death criterion in the Courts was felt to be a significant turning point in MAID eligibility assessments. Assessors were no longer required to define what constituted a reasonably foreseeable natural death, and this opened the door to more persons being eligible for MAID.

*The comfort level that our providers had in terms of assessing people, it shifted remarkably, it gave increased confidence. And so, after that ruling, we saw more people beyond a cancer diagnosis, so we saw more people with MS, we saw more people with Parkinsons, more with advanced osteoarthritis and we saw diagnoses of frailty. (P75)*

The fact that it was no longer about prognosis but about an illness trajectory made assessments easier. “*It’s more are you on a path of morbidity and illness that there’s going to be a complication that’s going to end your life at some point? So, I found that when that criterion was changed or removed, that actually made it a lot easier for me.” (P84)*

#### Stress of telling someone they are ineligible

A powerful incentive for getting these eligibility assessments right was the stress associated with deeming a person ineligible for MAID. Assessors spoke of a sense of relief they felt when getting a referral that was relatively straightforward. The more complicated cases were emotionally intense. Assessors were aware that patients did not enter into these eligibility assessments lightly and that they often experienced a sense of relief once they were deemed eligible. Participants told stories of being involved with complex cases and having to navigate the “*pushback*” *(P94)* that would come from the different people involved in the assessment. These assessment situations were described as even more emotionally taxing than the provision of MAID. In some situations, assessors had to withdraw from the assessment process to preserve their own mental health. “*We really try to work through it. Right? But it’s a lot of work and so when that’s starting to disrupt my sleep and affect my ability to interact with my kids in a motherly way, sometimes I have to just push things back.” (P94)*

Assessors expressed feelings of guilt and sadness over having to deem someone ineligible. Statements that ineligible persons made, or actions they took in response to the assessment, were imprinted on the assessor’s memory. One participant reflected on the response of an elderly client “*Who has the right to determine what amount of suffering I’m in in my own life? I’m 80, I’ve lost my sight, I’m in constant pain, I don’t feel like eating and I’m not eligible?“* This client went on to experience medical complications that resulted in a trajectory of suffering. Such statements by patients were particularly difficult for assessors when the primary reason for MAID was resolving suffering.

#### Finding a way to make someone eligible

When applicants did not meet eligibility criteria, there were still options for those who were determined and resourceful. “*So, it’s almost like you can find an assessor that will qualify you if you are a very resourceful, smart person*.” *(P67)* In some situations, persons would seek a third assessment and through it ultimately be successful in being deemed eligible. MAID coordinators might assist this process by screening potential assessors to ensure that they were well-versed in the criteria. “*We’re kind of protective of the patients and we try not to expose them to people that are going to say no. And so, we vet the physician or nurse practitioner beforehand. And just be, like, ‘Do you think that you’re going to find this patient eligible? Like, what are… what’s your understanding of the eligibility criteria’”? (P86)* In other cases, where it became apparent to the clinician that persons were determined to have MAID, the clinician might counsel them about the medical treatments they could refuse that would put them in a position of being eligible. For example, this nurse described the situation of a person who was living with a chronic condition that required ongoing intensive treatment that they were no longer willing to bear. Although she was unable find him eligible for MAID, she was able to outline a path by which he could become eligible should he choose to do so. “*If you decide, ‘I’m not going to, you know, take antibiotics for my urinary tract infections. I’m not going to do X, Y, or Z preventative care.’ I would then find him eligible.” (P94)*

#### Waiting periods and final consent

The safeguard that participants found most challenging was the ten-day waiting period and the need for patients to provide final consent prior to the injection of the medication. The biggest concern was that patients were taking MAID too early because they feared losing capacity. “*If you’ve got a certain disease or illness that could potentially incapacitate them, it’s distressing for them because then their choices get narrower and they may end up having to do MAID sooner than what they would want*.” *(P48)* Participants also described family who were concerned about providing pain medication because it might lead to the loss of the person’s ability to give consent. "*When a client is taking quite a bit of pain medication and they’re starting to be more and more drowsy I’ve heard questions from the family like, ‘Should I withhold medication? Because he’s comfortable but he’s not really responding.’ And so, they’re worried about that last-minute withdrawal of consent*." *(P71)*

Families could be deeply impacted if their family member had sought MAID but could not have it because they lost the capacity to give consent at the end. “*Half an hour before her MAID was to happen, she stroked. She was unresponsive and the family went crazy. They were, like, ‘Mom, mom, God, wake up. Mom, you have to be able to say it.’” (P40)* The consequences of this part of the legislation were perceived to be so detrimental to patients and families that some providers simply did what they felt to be the ethical thing to do. Participants witnessed instances of the waiting period being waived on a regular basis or of the final injection being provided even when the ability to give final consent had become questionable. However, it is important to note that in most instances these were seen to be ethical acts that were necessary in the case of inflexible safeguards that did not protect patients’ best interests.

### Anticipating Bill C-7

At the time that this data was collected, the intentions surrounding the new legislation Bill C-7 were publicly available but was still being debated in government. We did, however, ask participants about their perceptions of the impending new legislation. Overall, those who were acting as assessors and providers were more aware of the content of the changes and the subsequent implications than were those who primarily support patient care. Many of the latter had little to no knowledge of the changes.

Participants indicated that the timing of the changes was difficult when the systems and processes were just now settling into an equilibrium after the implementation of Bill C-14. Several assessors and providers were uncertain about whether they would continue to participate under the new legislation. Two primary concerns characterized this reluctance: the complexities that would characterize the assessment and provision of MAID and the lack of resources available to meet the needs of the newly eligible population whose natural death was not reasonably foreseeable.

#### New populations bring new complexities

Even though the criterion of foreseeable death had been struck down by the courts, and therefore realistically clients did not need to be at end-of-life under Bill C-14, participants felt that Bill C-7 would lead to whole new populations requesting MAID. Specific populations cited included those with mental health or psychiatric conditions, those living with chronic pain, and those who were living with disabilities. This was seen to be a momentous change by some. *At the point of which folks with mental health issues can have an assisted death, then that kind of changes everything for me. (P80)* Although Bill C-7 contained an embargo on assisted death for those whose underlying medical condition was psychiatric, participants knew that this embargo was time limited.

Participants further suggested that it might be difficult to assign persons to one of the two tracks available under Bill C-7 in light of their challenges with determining what constituted a reasonably foreseeable natural death under Bill C-14.

*I’m personally not super excited about having turned into a two-stream process (C-7). Logistically, it will be a problem because I think our hope was in removing the reasonably foreseeable death criterion would mean we don’t have to figure out what does reasonably foreseeable mean, but now we do. And I think that is even more challenging. (P83)*

Whereas the striking down of the reasonably foreseeable criterion by the Court had the effect of creating a more relaxed assessment climate, assessors were now going to be required to grapple with that criterion in a new manner that had significant implications for MAID applicants, as those without an imminently foreseeable death were required to apply under more stringent safeguards.

The complexity of cases that were anticipated under Bill C-7 had been foreshadowed under Bill C-14. These complexities included applicants with psychiatric conditions, who were disenfranchised from the healthcare system, or who were experiencing extreme pain for which no underlying cause could be found. Addressing these complex cases was going to require “*really having to dig deeply into their medical records and specialist reports to determine, you know, does their entire picture of medical concerns allow them to meet the eligibility criteria of a grievous and irremediable medical condition.” (P79)* When referring to those experiencing pain without a clinical diagnosis, one participant stated “*Those are the ones that keep me awake at night*.” *There’s a lot of things about C-7 that make me very uncomfortable.” (P92)* Another participant shared the challenges of working with this complex population over the phone as a coordinator.

*So, there are people who call with psychiatric issues and those can be very long and very difficult conversations because they don’t qualify and they feel they’ve been, I quote, ‘screwed by the system’ repeatedly. And I’ve had people say to me, ‘Well, then you’re forcing me to go find a gun or something.’ And I don’t accept that. I just say, ‘No. I’m not. I’m telling you what the law says.' And remembering that that’s a phone conversation and we don’t have any visual or physical cues. (*P89)

#### A cry for help not MAID

The conversations like the one described above were often perceived by healthcare providers as a cry for help rather than a request for MAID. “*What we’ll find, especially with, you know, C-7 on the table is that there’s a lot of people coming forward who aren’t really looking for MAID” (P92).* “*I’ve had so many conversations with people about wanting to pursue MAID and then when you have a conversation and you realize they don’t want to pursue it at all, you know, it’s other things that they’re crying out for*.” (P84) These needs might arise from effects of a devastating loss (e.g., spinal cord injury) or from a lack of basic needs such as housing, food, or social connection. Participants did their best to connect these individuals with necessary resources, but also recognized that such resources might not be available in a timely manner. For example, persons suffering from pain they considered debilitating might have to wait 6 months for a referral to a pain clinic. One option would be to fast-track services for clients who were applying for MAID, an option that could have numerous negative consequences. “*And we don’t want that to happen because we don’t want people to be arbitrarily asking about MAID in order to get the resources that they need. So, those are some of the concerns that our team has been talking a lot abou*t.” (P87) This then left the ethical question of whether MAID was an acceptable option when supports that would alleviate suffering existed but were not accessible. One participant pondered the fact that the waiting period for MAID under C-7 was shorter than the waiting period to access some specialist services in her area. “*Access to consultants is going to be a challenge. Access to consultants for people who actually have conditions that they’re looking to treat is a challenge, so I am anticipating problems in being able to access consultation services.”* (P83)

Bill C-7 was anticipated to bring new challenges during a time when healthcare providers were just getting settled with C-14.

*The legislation is moving faster than we have been able to create grounding for. We’re constantly behind the eight ball and constantly saying, “I’m sorry”, and constantly trying to support patients while also trying to figure it out. So, we’re happy to do this work that needs to be done but it’s going to be challenging. (*P86)

Over time, nurses had learned the inherent complexities associated with the original legislative conditions of Bill C-14, and therefore were expressing their reservations about Bill C-7 from a highly informed and critically reflective perspective. Participants understood the potential pitfalls within the new legislation and were aware that they would need to work out how to safeguard patients and ensure that the system was organized to respond appropriately to the evolving demand.

## Discussion

This study has several limitations that should be considered when applying the findings. The majority of participants were from geographic areas within Canada where MAID had already made significant inroads, as evidenced by higher prevalence. Therefore, not all of these findings will necessarily be transferable to geographic contexts where the prevalence of MAID is lower. Further, the majority of our participants were in agreement with the idea of MAID, at least in theory^2^, and so the views should not be considered indicative of those opposed to MAID. Finally, our sample included more registered nurses than nurse practitioners when nurse practitioners are the only nurses in Canada permitted to assess for and provide MAID. Nevertheless, findings have important implications for system level considerations relevant to the implementation of Bill C-7. Notably, these concern the cultural calibration that is required after each substantive change to MAID law and the complex challenge of navigating siloed and inaccessible healthcare systems when the decisional stakes are high. 

### Cultural calibration

Evidence from other countries where assisted death has been legal for a number of years suggest that calibration occurs as the system, and persons within the system, adjust to incorporating assisted death as part of their practice [32–35]. Hamarat, drawing upon an ethnographic study of assisted dying in two countries, suggested that over time “aid-in-dying” becomes an “institution” in itself that is “framed, bound, and structured by norms.” ([36] p. 1) These “normativities” are structured through legal and regulatory frameworks, organizations, discursive practices, technical and bodily practices, and relationships. The influence of relationships in developing these norms was evident in a study conducted in Quebec prior to the legalization of assisted death that explored nurses’ intention to be involved. An important factor in nurses’ decisions was the opinions of their colleagues, a form of social calibration to a morally complex act [37] .

Findings from this study described a similar process of social calibration. Nurses became more comfortable with MAID procedures, but more importantly, a culture of openness and trust developed which in turn led to decreased stress in the work environment. Some referred to this process as the normalization of MAID. However, this does not imply a ‘slippery slope’ in which MAID was simply being extended to more and more people with little thought of consequences. Rather, normalization referred largely to system processes that slowly became calibrated to the needs of patients and families and in which participants adjusted themselves to the moral demands. Participants also suggested that there was a component of the act that should never feel normal and they continued to feel the emotional impact, similar to what Flemish nurses reported many years after the legalization of euthanasia in Belgium [38]. These findings of calibration stand in contrast to our earlier interviews with nurses, conducted shortly after the legalization of MAID, in which participants described an unprepared healthcare system that left them with few practice supports to do the morally complex work that was required with this new legal responsibility [19, 25, 27].

Participants in this current study were anticipating that a similar calibration would be required under Bill C-7, including new moral questions. Could they see themselves participating in a MAID process where natural death was no longer reasonably foreseeable? Could they participate in the MAID process when the means to relieve suffering existed but not might be accessible? Would the healthcare system be prepared for it? Would there be enough assessors and providers willing to do this work? Participants were once again anticipating having to do the hard moral work of questioning their involvement in MAID.

The implementation of Bill C-7 will likely necessitate recalibration both at the social and systems level. An important focus must be on influencing the development of norms toward high quality practice within a reflective ethical context. Healthcare providers involved with MAID consider many aspects beyond the legality of eligibility and safeguards; their decisions are influenced by their own values and beliefs in each clinical encounter, and with varying degrees of self-knowledge [39, 40]. As such, working within functional and supportive teams is an important strategy to mitigate the effects of individuals working in isolation [19, 20, 41]. The development of moral communities of practice, supported by robust policy [42] are key to developing high quality, ethical norms. The importance of this type of community was evident in our data when participants described how interpretation of eligibility criteria was a gestalt and when they described the normative expectations that shaped eligibility assessments.

### Navigating a siloed and inaccessible systems

This, however, leads to the question of how to set up services. Do we create one more clinical program within an already deeply siloed and disconnected system, and if so, how might that new program be successfully integrated? The implementation of Bill C-7 provides a unique opportunity to improve upon the implementation process that occurred under Bill C-14. There is robust evidence that high quality MAID care depends upon skillful communication, knowledge of suffering at end-of-life, and resources that acknowledge the time required to do the work well [20, 41]. In Canada, a number of approaches have been used to deliver MAID care: from well-developed clinical programs that span the mandate of research, education and practice to assessors and providers who work alone and travel large geographic areas to provide services. Given Canada’s diverse sociocultural and geographic nature, there will be no one-size-fits-all approach.

Participants in this study explored the possibilities and drawbacks of integrating MAID and palliative care programs; although, this was a contentious proposal because of what some would argue are different philosophies of care. Yet despite these philosophical differences, MAID providers and palliative care providers serve the same population so an approach that puts patients at the centre of systems would be desirable from the central standpoint of the patient who is looking for the best possible end-of-life. Further, palliative care clinicians are highly skilled in addressing issues of suffering, in improving quality of life, and in conducting care planning conversations which are all critically important aspects of MAID. However, among the palliative care community in Canada there remain significant concerns about the insufficient availability of palliative care which leads to inequities in patient choices [14]. Participants in this study cited significant equity concerns about access to both MAID and palliative care and highlighted the political tensions around funding allocations. As explained in the background to this paper, the evidence about the degree to which patients have access to palliative care in Canada is difficult to interpret.

One way to inform this debate is to examine the trajectories of other countries where assisted death has been legal for a decade or more to explore how these relationships have evolved and to determine what the effect has been on palliative care. Evidence to-date has indicated that where euthanasia is legal, the relationships between euthanasia and palliative care range from conflicted to cooperative [43]. Further research is required to better understand the factors that influence these relationships. However, evidence to-date has also indicated that palliative care improves after the implementation of assisted death [44]. But, these findings need to be contextualized within the policy commitments of those countries. For example, in Luxembourg, at the same time that assisted death was decriminalized, palliative care was declared a basic patient right, universal access to palliative care was funded, and mandatory targets were set for services [44]. Canada’s approach has been much softer with the development of a framework [17] and some targeted funding for improvement. We do not yet know what the real effects of that Canadian approach have been.

Bill C-7, however, brings new complexities that means that system integration between MAID and palliative care is a less viable approach. Within the two-track system where reasonably foreseeable natural death is the determinant of what track a person gets assigned, a number of these MAID applicants will not be incurably ill and will require expertise from social and mental health services. For a person considering MAID, whether death is foreseeable or not, the most important healthcare intervention is relational and dialogical in nature. Best practices entail a relational interaction between the applicant and a knowledgeable healthcare provider in which the suffering story can be shared, mitigation strategies can be considered, and the request is considered within the constellation of a life story [39]. In countries where MAID is provided primarily through the primary care system, long term relationships between physicians and patients support this best practice. However, in Canada the system often depends upon assessors and providers who have no prior knowledge of the patient. In the context of centralized coordination services being the primary means of delivery, different strategies must be enacted to ensure this relational and dialogical approach. Further, because of the requirements of C-7, these persons must have superb relational and communication skills; extensive knowledge of the health and social care system to ensure that patients know what is available to them to alleviate their suffering; exceptional advocacy skills to help persons get access to those services; and sufficient time and remuneration to do this complex work.

In jurisdictions where MAID services are centrally coordinated, some of the participants in this study were already performing a role that met these criteria. These were the nurse coordinators of MAID services. As the first contact for the public, they had learned to screen and navigate clients to ensure that they did not enter the eligibility process if they were likely to be disappointed. They had learned to navigate clients to services that would help to alleviate their suffering. Further, they were performing an advanced practice role as they provided public and healthcare provider education, created policies, and served as connectors among the many persons involved in a MAID death. Creating such systems of navigation is an important goal cited in the National Framework for Palliative Care in Canada [17]. The model of care that has been adapted for the Canadian context exists but significant systems calibration is required to expands its scope to meet the complexity required of Bill C-7.

## Conclusions

Canada has made significant inroads since 2016 in adjusting the healthcare system to meet the needs of patients and families seeking a MAID death. The process of MAID has become increasingly normalized even as the gravity of the event has retained its emotional impact. MAID assessors are developing consensus about clinical application of the legal criteria and nurses are finding ways to support a patient-centered approach to care. However, increased demand for MAID, and a rise in the complexity of clients requesting MAID, is putting significant strain on the system. Bill C-7 is raising complex new issues, including moral concerns about extending MAID to new populations, access challenges to the services required to alleviate the suffering of this population, further increases in workload, and a potential shortage of assessors and providers willing to stay engaged with MAID work. Such challenges may be partially met through the development and expansion of the coordinator/navigator role. Such individuals will require superb communication and advocacy skills, extensive knowledge of the health and social care system, and sufficient time to do this complex role well.

These findings provide important insights into the practical challenges facing health systems as they seek to create best practices around MAID care. They provide insights for policy and healthcare decision-makers as health systems adapt to the evolution of MAID through Bill C-7. While such insights may be specific to the immediate situation in Canada, they will have relevance for other countries considering legalizing assisted death. Finally, they make apparent the exceptionally important perspective that nurses bring to the development of systems, strategies and best practice approaches as we expand the available options for all persons facing end-of-life care considerations.

## Supplementary Information

**Additional file 1.**

## Data Availability

The datasets used and/or analysed during the current study are available from the corresponding author on reasonable request.

## Abbreviations

MAID: Medical assistance in dying
NP: Nurse practitioner

## Footnotes

1 In Canada faith-based facilities are allowed conscientiously objecting status based upon previously existing agreements between the faith-based system and the public system. This has created a number of barriers for patients and so anything that helps to overcome those barriers is perceived as a kindness.

2 Participants will state that they agree with MAID but are less certain about whether they could administer it or choose it for themselves.
