# Supplementary material for: Navigating medical assistance in dying from Bill C-14 to Bill C-7: a qualitative study
Source: BMC Health Serv Res. 2021 Nov 4;21:1195. doi: 10.1186/s12913-021-07222-5 (PMC8567982; doi:10.1186/s12913-021-07222-5)
Supplement: Supplementary file 1 — Additional file 1. [file 12913_2021_7222_MOESM1_ESM.docx]

**Strategies to Relieve Suffering at End-of-Life**

**Nurse Participant Semi-Structured Interview Guide**

**Note to Interviewer:** Follow the flow of the interviewee but be sure to probe around all applicable areas

Please begin by telling me more about your current role in relation to MAiD and/or palliative care.

***Probes around Suffering***

- I want you to think about when you first started practicing your profession. Have your ideas of suffering changed from then to now? If so, how?
- Can you tell us about your experiences of caring for those who experience suffering at end of life? What have you learned about alleviating suffering?
- Suffering and MAiD: One of the criteria for MAiD is that someone must be *enduring physical or psychological suffering that is intolerable and cannot be relieved under conditions that they consider acceptable*. Can you tell me more about the types of suffering you see in individuals who are considering MAiD? What have you learned about assessing the suffering criteria as part of the MAid eligibility process? Can you imagine a time when you might consider someone ineligible based upon your assessment of their suffering? In your experience, does the process of considering or receiving MAiD cause suffering for anyone involved in the process?

***Probes around MAiD Care and Communication***

- What is it like to work with patients who are in the initial stages of considering MAiD? What have you learned about handling those initial inquiries?
- Some regions in Canada are introducing MAiD as one of the end of life care options as part of advanced care planning. How does that work in your part of the country?
- What have you learned about good communication during MAiD?
- What have you learned about good care during MAiD?

***Probes around MAiD Eligibility and Safeguards***

- Can you tell us about your experiences with determining MAiD eligibility and safeguards?
- What is working well in your opinion? What is working not so well? (ask them to describe a complicated assessment)
- How do you think Bill C-7 is going to change your MAiD work, if at all?

***Probes around Systems Interaction***

- How are palliative care and MAiD organized in your region? What have been the advantages and disadvantages of that approach?
- How is the organization of MAiD/Palliative care working in your jurisdiction? What is working well and what improvements need to be made?
- What is the relationship like among the palliative care and MAiD teams/providers?
  - If they talk about how they interact how it works together….
  - Can you tell me how those relate?
  - Probe around structure
  - Do they have common staff? Do they report to different people? How do you navigate determining eligibility?
- What changes, if any, have you seen to palliative care since the implementation of MAiD?

***Death***

1. What have you learned about death in the context of MAiD?
   1. What has gone really well and has felt like a “good death”
   2. How would you characterize a “good death”
   3. What experiences have not gone as well and would make you consider them to be a less successful death?

***Provider Well-Being/ Competency***

1. What have you learned as a person about being involved with MAiD/PC, or not involved, over time?
2. What kinds of supports are available, or should be available, to staff who are involved with MAiD services?
3. What kinds of things have you learned about self-care in the context of being involved with MAiD?
4. What education/competencies do you think nurses should have to be involved in MAiD? How do they achieve those competencies in your area?

***Wrap up Question***

What have we forgotten to ask you about that you think we should know?
